# Polidocanol Sclerotherapy Combined with Transarterial Embolization Using *n*-Butyl Cyanoacrylate for Extracranial Arteriovenous Malformations

**DOI:** 10.1007/s00270-017-1855-2

**Published:** 2018-02-07

**Authors:** Akira Kitagawa, Takahiro Yamamoto, Nozomu Matsunaga, Mayako Yamaji, Shuji Ikeda, Yuichiro Izumi, Makiyo Hagihara, Toyohiro Ota, Tsuneo Ishiguchi

**Affiliations:** Department of Radiology, Aichi Medical University, 1-1 YazakoKarimata, Nagakute City, Aichi 480-1195 Japan

**Keywords:** Embolization, Sclerotherapy, Arteriovenous malformations, Polidocanol, NBCA

## Abstract

**Purpose:**

To assess the safety and effectiveness of polidocanol sclerotherapy combined with transarterial embolization using a liquid adhesive agent (*n*-butyl cyanoacrylate, NBCA) for treatment of extracranial arteriovenous malformations (AVMs).

**Materials and Methods:**

Twenty-three patients with symptomatic AVMs in the head and neck (6), upper (7) and lower extremity (10) with a mean age of 42 years (range 4–74) treated with polidocanol sclerotherapy were retrospectively assessed. AVMs were classified according to the angiographic morphology of the nidus. There were 2 type I, 6 type II, 6 type IIIa and 9 type IIIb. Arterial embolization using NBCA was performed to reduce arterial flow before sclerotherapy. Polidocanol mixed with contrast material or carbon dioxide was delivered by percutaneous direct puncture.

**Results:**

Treatment was successfully performed in all patients. In the mean follow-up period of 38 months, symptoms resolved or improved in 20/23 patients (87.0%). AVMs were devascularized 100% in 2 patients, 76–99% in 13, 50–75% in 7 and < 50% in 1. More than 50% devascularization was seen in 22 patients (95.6%). Two (8%) patients had complete remission, 17 (74%) had partial remission and 3 (13%) had no remission. There was no aggravation. Treatment was considered effective (complete and partial remission) in 20 patients (87.0%). Minor complications including localized arterial thrombosis (2) and spontaneously healing skin ulcer (1) were seen in 2 patients (8.7%). There were no major procedure-related complications.

**Conclusion:**

Polidocanol sclerotherapy combined with transarterial embolization using NBCA is safe and effective for treating extracranial AVMs with an acceptable risk of minor complications.

## Introduction

Congenital arteriovenous malformation (AVM) is a high-flow vascular malformation composed of arteries and veins that directly communicate through arteriovenous fistulae. AVMs cause symptoms including skin blush, swelling, bruit, pain, ulceration, bleeding and malfunction. Large AVMs with high flow into the venous system may lead to volume and pressure overload and subsequent heart failure [1]. Complete surgical resection of AVM is rarely possible, except when it is small and localized in a surgically accessible area, and partial excision of AVM is followed by recurrence, usually with a worsened condition [2]. Embolization and sclerotherapy have been performed as less-invasive treatments of AVMs using various embolic materials (particles, liquid agents or coils) and sclerosing agents (ethanol, sodium tetradecyl sulfate or ethanolamine).

Transarterial embolization using *n*-butyl cyanoacrylate (NBCA) has been the most common technique to treat AVMs. NBCA alone, however, is frequently insufficient to achieve complete occlusion of the AVM, especially when multiple fistulas are present.

The clinical experience with ethanol embolization achieved by catheterization or direct puncture has been favorable [3–5] despite the pain associated with ethanol injection, while minor and major complications have been relatively frequent [1, 5]. Transarterial embolization using *n*-butyl cyanoacrylate (NBCA) combined with ethanolamine oleate (EO) sclerotherapy has been reported as a safe and effective treatment with an acceptable risk of minor complications [6], but EO solution with the proper concentration is available only in limited countries. Polidocanol is another sclerosant widely used for sclerotherapy of lower limb varices [7]. Polidocanol has been used for venous malformation in some investigations [8, 9], but infrequently for AVM, and no study has reported a large number of cases treated with polidocanol. This prompted us to conduct the present study in which the safety and efficacy of polidocanol sclerotherapy in combination with transarterial embolization using NBCA were evaluated for the treatment of extracranial AVMs.

## Materials and Methods

### Patients

The study consisted of a retrospective review of patients with AVMs treated with polidocanol sclerotherapy combined with transarterial embolization using NBCA at our institution for the past 6 years between July 2010 and November 2016 (Table 1). This retrospective study was approved by the institutional review board. There were 23 consecutive patients, 7 males and 16 females with the mean age of 42 years (range 4–74 years). There were a total of 29 treatment sessions. The most common anatomical site of the AVMs was the lower extremity (*n* = 10), followed by head and neck (*n* = 6) and upper extremity (*n* = 7). The symptoms/signs included pain, swelling, bleeding and skin ulceration. All patients had more than one symptom/sign. The nature of the disease was explained to the patients, and informed consent was obtained as to the options for treatment and potential risks and benefits of embolization and sclerotherapy.

**Table 1:** Characteristics and results of 23 patients with AVMs

Pt. no./sex/age	Location	Type	Symptoms	No. of procedures	No. of NBCA injection	Total amount (mL) and % of NBCA/Lip	No. of puncture for Pol injection	Total amount (mL) and % of Pol/CM	Complications	Devascularization (%)	FU months	Outcome	Clinical sequel
1/F/38	R Thigh	II	Pain, swelling	1	2	2.6 (25)	2	5.0 (80)	None	100	46	CR	
2/F/8	R Temple	IIIa	Pain, swelling	1	1	0.5 (20)	1	5.0 (80)	None	76–99	44	PR	
3/F/41	L Foot	IIIb	Ulcer, bleeding	1	1	0.7 (25)	3	6.0 (67)	None				
				2	5	1.6 (25)	3	12.0 (67)	Arterial thrombosis	50–75	19	PR	
4/M/51	L Hand	IIIb	Bleeding	1	2	1.6 (25)	1	6.0 (67)	None				
				2	2	1.3 (28.6)	1	4.0 (67)	Arterial thrombosis, Ulcer	76–99	24	PR	
5/F/63	L Forearm	II	Pain	1	4	2.0 (25)	2	5.4 (67)	None				
				2	2	1.3 (28.6)	2	7.2 (67)	None	76–99	49	PR	
6/M/66	L Arm	II	Heart failure	1	3	10.0 (20, 14.3)	2	5.3 (67)	None	<50	19	NR	Deceased^a^
7/F/58	R Hand	IIIa	Pain	1	3	0.6 (25)	2	5.0 (67)	None				
				2	2	0.5 (25)	1	4.0 (67)	None	50–75	52	NR	Recurrence
8/F/74	L Foot	IIIa	Ulcer, pain	1	1	0.6 (25)	1	6.0 (67)	None	50–75	18	PR	
9/F/22	R Eyelid	IIIa	Swelling	1	4	1.8 (25)	1	6.0 (67)	None	76–99	63	PR	Resected^b^
10/F/4	L Finger	IIIb	Swelling	1	1	0.3 (25)	3	3.0 (67)	None	76–99	60	PR	
11/F/46	L Knee	IIIa	Pain	1	1	0.2 (25)	1	2.0 (67)	None	76–99	63	PR	
12/M/47	Nose	IIIa	Bleeding	1	2	0.3 (20)	2	4.0 (67)	None	76–99	30	NR	Rebleeding
13/F/23	L Masseter	IIIb	Pain, swelling	1	3	0.7 (25)	2	6.0 (67)	None	76–99	59	PR	
14/M/27	L Foot	IIIb	Pain, swelling	1	5	1.4 (25)	1	9.5 (67)	None	76–99	42	PR	
15/F/63	R Foot	IIIb	Bleeding	1	1	0.4 (25)	1	6.0 (67)					
				2	1	0.4 (25)	1	6.0 (67)					
				3	1	1.3 (33)	1	5.0 (67)	None	50–75	21	PR	
16/F/71	L Forearm	II	Swelling	1	3	0.8 (25)	2	4.0 (80)	None	76–99	34	PR	
17/F/38	R Foot	IIIb	Pain, swelling	1	1	0.8 (25)	1	7.5 (80)	None	76–99	48	PR	
18/M/50	L Lip	IIIb	Pain	1	3	0.7 (25)	2	4.0 (75)	None	50–75	31	PR	Resected^b^
19/M/13	L Thigh	I	Pain, swelling	1	2	1.1 (33, 25)	1	5.5 (73)	None	50–75		PR	
20/F/27	L Foot	II	Swelling	1	2	3.1 (25)	1	2.8 (80)	None	76–99	36	PR	
21/M/49	Forehead	I	Bleeding, swelling	1	2	1.2 (25)	1	6.0 (67)	None	100	24	CR	
22/F/39	R Hand	IIIb	Pain	1	4	5.9 (25)	1	1.9 (80)	None	76–99	12	PR	
23/F/73	R Foot	II	Pain, swelling	1	4	3.2 (25)	1	Foam 15 (Pol:Lip:CO2= 2:1:8)	None	50–75	12	PR	

*Lip* lipiodol, *Pol* polidocanol, *CM* contrast-enhanced medium, *CR* complete remission, *PR* partial remission, *NR* no remission

^a^Not procedure-related

^b^For cosmetic purposes

### Diagnostic Imaging

AVMs were diagnosed based on both the clinical symptoms and imaging data. Lesions were first assessed by physical examination, ultrasonography, dynamic contrast-enhanced magnetic resonance (MR) imaging or computed tomography (CT). MR imaging was performed with 1.5-Tesla units (Avanto SQ; Siemens AG, Munich, Germany). Gadolinium-enhanced T1-weighted three-dimensional gradient echo imaging was performed with 0.2 mL gadolinium contrast medium (0.5 mmol/mL) per kilogram of body weight at a rate of 1.0–1.5 mL/s and a subsequent 20-mL saline flush using a power injector. The timing of image acquisition was decided by 0.1-mL bolus test injection. CT examinations were performed with 64 multi-detector row units (Aquillion 64; Toshiba, Tokyo, Japan or LightSpeed VCT; GE Healthcare, Waukesha, USA). Dynamic contrast-enhanced CT imaging was performed after the intravenous injection of 2.0 mL/kg of body weight at a rate of 3.0–3.5 mL/s using a power injector. The scan was started with the trigger by attenuation of the aorta. 3D CT angiographic reconstruction was performed with a workstation (Ziostation 2 ver 2.x; Ziosoft, Tokyo, Japan).

### Angiographic Classification

In all patients, selective catheter angiography was performed to obtain detailed anatomical and hemodynamic information. Digital angiography systems with flat-panel detectors (Artis zee and Artis dbA twin; Siemens AG, Munich, Germany) were used. Sequential digital subtraction angiographic images were transferred as movie files and stored in a server system (Kada-View; Photron Medical Imaging, Tokyo, Japan).

Two board-certified interventional radiologists reviewed the initial angiography and classified the AVMs by consensus based on the modified angiographic classification of Cho et al. [10]. The AVMs were classified into 4 types according to their angiographic morphologies.

### Procedures

#### Arterial Embolization

Two preteen patients were treated under general anesthesia and all others under local anesthesia. First, a selective angiographic study using a 4-French catheter was conducted, and then a microcatheter advanced to the feeding arteries of the AVM. The selection of the type of microcatheter was based on the size and tortuosity of the feeding arteries. The most commonly used microcatheters were 2.0-French (Progreat Alfa; Terumo, Tokyo, Japan) and 1.9-French (Progreat Sigma; Terumo, Tokyo, Japan). Microcatheters with a coaxial system of 2.7-French (Renegade Hi Flo; Boston Scientific, Natic, USA) and 1.6-French (Carnelian Marvel; Tokai Medical Products, Aichi, Japan) were used when necessary. The flow and anatomy of the AVM were confirmed by digital subtraction angiography (DSA) with manual injection of contrast material (Visipaque 270; Iodixanol, DaichiSankyo Co. Ltd, Tokyo, Japan).

Mixture of NBCA (Histoacryl; B Braun Aescloup, Tuttlingen, Germany) and iodized oil (Lipiodol; Andre Guerbet, Aulaysous Bois, France) was used as an embolic agent for transarterial embolization with a microcatheter. The microcatheter tip was inserted as closely as possible to the nidus beyond the normal branch. Based on the position of the catheter tip and flow speed, NBCA and lipiodol were mixed at a volume ratio of 1:3–1:5. A 1-mL Luer-Lock syringe was filled with the NBCA–lipiodol mixture, and after flushing the microcatheter with 5% dextrose solution, the mixture was injected while monitoring the DSA images. The injection was stopped and the microcatheter was withdrawn after flow stasis of the feeding artery and retrograde filling of the mixture toward the microcatheter tip were confirmed. “Plug and push” technique was often used; it involves the creation of a glue plug around the catheter tip, followed by progressive filling of the AVM nidus [11]. When an AVM had multiple feeding arteries, embolization was repeated via other feeders until the entire flow of the AVM became slow. The aim of embolization was not complete disappearance of flow in the nidus, but slowing down the flow to perform an effective sclerotherapy in the following session.

#### Sclerotherapy

Either in the same treatment session or independently following transarterial glue embolization, sclerotherapy using polidocanol was performed. Sclerotherapy was not performed when the nidus was filled with glue completely, and the cases with glue embolization only were excluded from this study. Catheter angiography was performed in all cases before and after sclerotherapy. Polidocanol solution of 3% concentration (Polidocasklerol 3%; Kreussler, Wiesbaden, Germany) was mixed with contrast material (Visipaque 270) at a volume ratio of 2:1–4:1. When a larger volume was required, polidocanol was mixed with iodized oil and carbon dioxide at the volume ratio of 2:1:8 using a three-way stopcock and pumping for foam sclerotherapy [9]. Injection of polidocanol was done by direct puncture into the fistulous point. When an AVM was located in the head and neck region or extremities, direct puncture was usually performed with a 23-, 24- or 27-G needle under road-map fluoroscopy or ultrasonography guidance. After trial injections of diluted contrast medium mixed with saline at a volume ratio of 1:1 in order to confirm the flow in AV shunts and draining veins and to estimate the volume of the sclerosant, polidocanol was injected under DSA monitoring until the AVM nidus became opacified. For AVMs in the extremities, proximal compression with a pressure cuff was applied to improve stasis of the sclerosing agent and prevent it from flowing into normal veins. For AVMs at the superficial face or scalp, manual compression of the draining vein was applied when possible. The duration of compression was 15–30 min, after which the needle was aspirated and removed. Injected volume of polidocanol was limited not to exceed 2 mg/kg body weight per session. Anti-inflammatory drugs or corticosteroids were administered for a few days after the procedure.

### Follow-Up

**Fig. 1:**
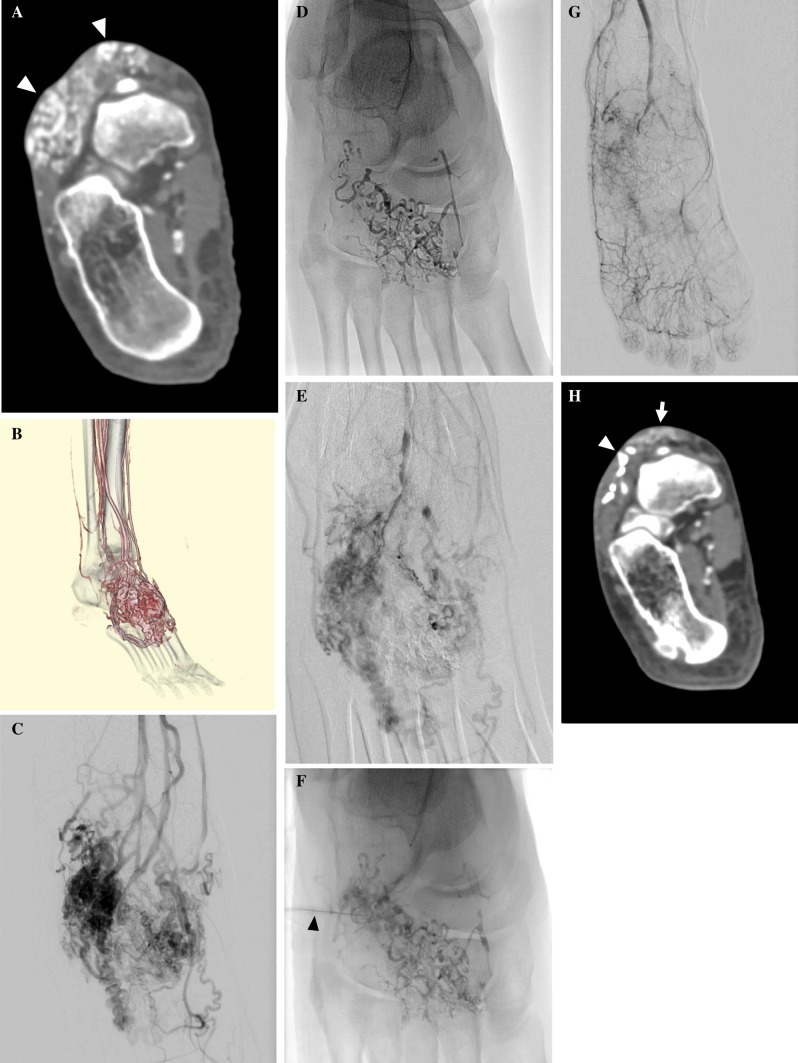
A 38-year-old woman with type IIIb AVM in the dorsum of the right foot with pulsating pain and swelling (Patient No. 17). **A** Contrast-enhanced CT shows a subcutaneous AVM with dilated abnormal vessels (arrowheads). **B** 3D-CT angiography demonstrates an AVM with dilated feeding arteries and draining veins. **C** Arteriography (frontal projection) demonstrates multiple feeders arising from the dorsal and plantar arteries and dilated draining veins. **D** Radiography after transarterial embolization from a feeding artery shows NBCA/lipiodol casts. **E** Arteriography after NBCA embolization demonstrates reduced flow of AV shunts. **F** Remaining AV shunts were punctured using a 27-gauge butterfly needle (arrowhead), and polidocanol sclerotherapy was performed under proximal compression using a tourniquet. **G** Arteriography after embolization and sclerotherapy shows almost complete exclusion of AVM. **H** Contrast-enhanced CT 3 years after treatment shows some remaining NBCA/Lipiodol casts (arrowhead) in the AVM and improvement in soft tissue swelling with shrinkage of subcutaneous veins (arrow). The patient has had no recurrence of pain in the long-term after the treatment. The therapeutic outcome was PR

**Fig. 2:**
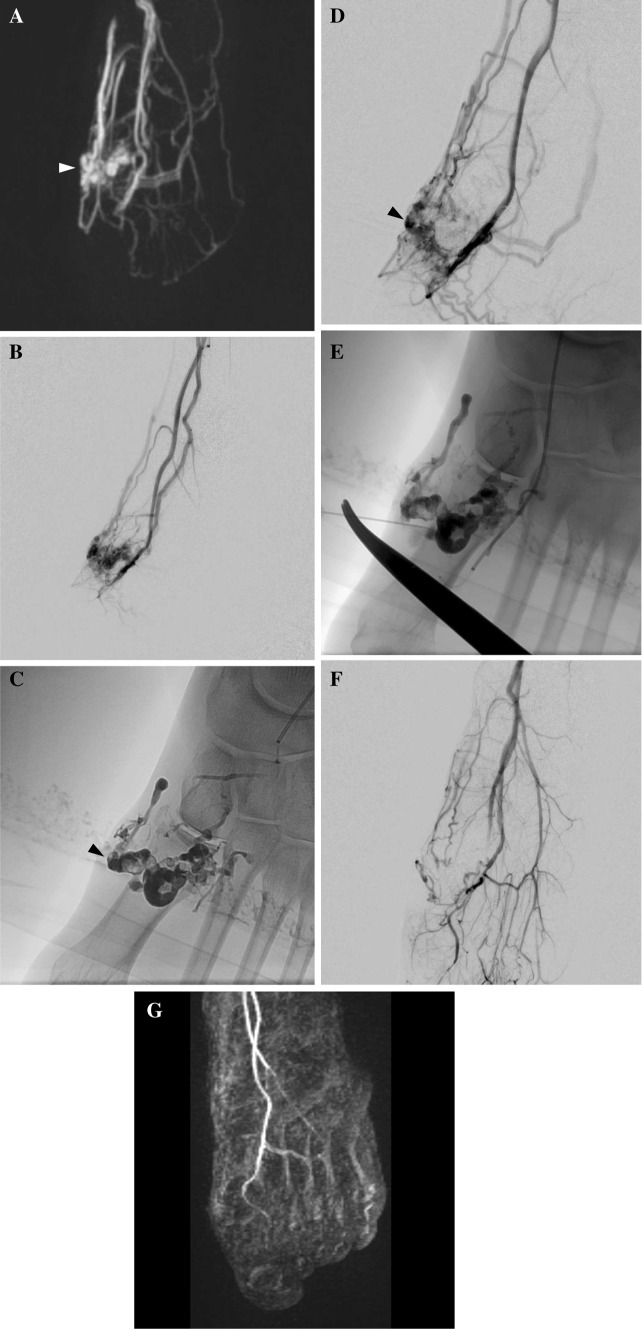
27-year-old woman with type II AVM of the left foot sole with worsening swelling (Patient No. 20). **A** Maximum intensity projection (MIP) image of coronal contrast-enhanced MR angiography shows an AVM (arrowhead) with dilated feeding arteries and draining veins in the left foot internal sole. **B** Angiography demonstrates an AVM with multiple feeders arising from dorsal artery and plantar arteries and draining veins. **C** Radiography showing NBCA/lipiodol casts (arrowhead) after transarterial embolization of 2 feeders. **D** Angiography after NBCA embolization shows reduced flow of AV shunts (arrowhead). **E** Remaining AV shunts were punctured using a 24 gaugeneedle, and sclerotherapy was performed by injecting polidocanol mixed with contrast medium under proximal compression using a tourniquet. **F** Angiography after embolization and sclerotherapy shows complete exclusion of AVM. **G** Contrast-enhanced MR angiography eighteen months after treatment shows complete exclusion of the AVM. The patient has had no recurrence, and the symptom has resolved. The therapeutic outcome was PR

Any immediate or delayed complications or changes in symptoms were closely looked for in all patients. Initially, this was done in hospital and then on an outpatient basis at 1, 3, 6 and 12 months. Post-treatment CT or MRI examinations were undertaken at 3–6 months after the procedure (Figs. 1, 2). Additional embolization or sclerotherapy was recommended if symptoms remained bothersome and persistent AVM was evident on follow-up imaging studies.

### Definitions of Outcome

The therapeutic outcomes were assessed by the two board-certified interventional radiologists based on the clinical response and degree of devascularization at angiography or CT/MRI.

Complete remission (CR) was defined as complete resolution of clinical symptoms and signs, associated with 100% devascularization of AVMs at angiography or contrast-enhanced CT. Partial remission (PR) was defined as complete resolution or some improvement in clinical symptoms and/or signs, with 50–99% AVM devascularization. No remission (NR) was defined as no change in clinical symptoms and signs, with less than 50% devascularization. Aggravation was defined as a worsening of clinical symptoms and signs, regardless of the degree of AVM devascularization.

CR and PR were considered to be effective therapeutic outcomes. For some patients requiring multiple treatment sessions, the analysis included clinical assessment after the last treatment session. Death, permanent adverse sequelae, need for major therapy and prolonged hospitalization exceeding 48 h were defined as major complications. Minor complications were defined as any nonpermanent adverse sequela, such as transient skin injury that healed spontaneously. Technical success in embolization and sclerotherapy procedures was defined as a successful catheterization and a subsequent partial or complete obliteration of the lesion. The therapeutic outcome was assessed at 3–6 months after the procedure.

### Statistics

Statistical analysis using Fisher’s exact test to the 4 × 2 table (Table 2) was performed to determine the relationship between the type of AVM and therapeutic effect. Statistical analyses were conducted using SPSS software (IBM SPSS Statistics version 22; IBM, New York, USA), and *p* values of less than .05 were considered significant.

**Table 2:** Therapeutic outcome of AVMs according to angiographic types

Type of AVMs	No. of patients	Treatment results			Effective cases (cure and PR)	Fisher’s exact test
		CR	PR	NR		
I	2 (9%)	1	1		2/2 (100%)	
II	6 (26%)	1	4	1	5/6 (83.3%)	
IIIa	6 (26%)		4	2	4/6 (66.7%)	
IIIb	9 (39%)		9		9/9 (100%)	
Total	23	2 (9%)	18 (78%)	3 (13%)	20/23 (87.0%)	*p* = 0.2987

*CR* complete remission, *PR* partial remission, *NR* no remission

## Results

Treatment was successful technically in all patients. NBCA was injected into a mean 2.0 arteries per case (range 1–5). The mean volume of injected NBCA and lipiodol mixture was 0.7 mL (range 0.2–4.0 mL) per injection. In all patients, polidocanol sclerotherapy was performed by direct puncture. The mean number of punctures was 1.7 times per case (range 1–3 punctures), and the mean volume of injected polidocanol and contrast medium was 5.5 mL (range 2.0–12.0 mL) per case. Polidocanol foam using carbon dioxide and iodized oil was used in only one patient, with the injected volume being 15.0 mL with one puncture. Multiple treatment sessions were performed for 5 patients; three procedures were performed in one patient, and two procedures in the others. Patients were followed up for an average period of 38 months (range 12–46 months) after their treatments (Table 1).

### Therapeutic Outcome

The symptoms subsided or improved in 20/23 patients (87.0%): Pain disappeared in 2/13 (15.3%), improved in 10/13 (76.9%) and did not change in 1/13 (7.7%). Swelling disappeared in 2/12 (16.7%) and improved in 10/12 (83.3%). Bleeding disappeared in 2/5 (40.0%) improved in 2/5 (40.0%) and did not change in 1/5 (20.0%). Skin ulcer improved in 2/2 (100.0%). AVMs were devascularized 100% in 2 patients, 76–99% in 13, 50–75% in 7 and < 50% in 1. More than 50% devascularization was seen in 22 patients (95.6%). Polidocanol sclerotherapy combined with transarterial embolization was assessed as effective (CR or PR) in 20 patients (87.0%): Two (9%) patients had CR, 18 (78%) had PR and 3 (13%) had NR. There was no aggravation.

Two patients (Patient No. 9 and 18) underwent plastic surgery for cosmetic purposes 3 months each after their treatments, when their original symptoms improved after the procedures.

The details of 3 patients with NR were as follows. Rebleeding occurred in one patient (Patient No. 12) 26 months after the treatment and was treated by NBCA embolization with no further bleeding. Recurrent pain occurred in another patient (Patient No. 7) but is being managed conservatively and will be considered for further treatment if the pain worsens. Another patient (Patient No. 6) died 19 months after treatment. He had a huge AVM in his left whole arm with severe heart failure already at treatment. The treatment in his case was performed as palliative therapy, and there were no procedure-related complications.

There were 2 type I, 6 type II, 6 type IIIa and 9 type IIIb. Treatment was considered effective (CR and PR) in type I: 2/2 (100%), type II: 5/6 (83%), type IIIa: 4/6 (67%) and type IIIb: 9/9 (100%). There was no significant difference in the effectiveness of treatment according to AVM types in the 4 × 2 table (*p* = 0.2987) (Table 2).

### Complications

Transient local pain, erythema and swelling due to inflammatory reaction with phlebitis were seen after the procedures in almost all patients. Minor complications including 2 localized arterial thrombosis and 1 self-healing skin ulcer were seen in two patients (8.3%). The arterial thromboses in two patients were found on the final angiogram after using a pressure cuff at the lower leg to reduce flow. One patient (Patient No. 3) had the arterial thromboses in her anterior tibial artery at the end of the session in her second treatment. 240,000 units of urokinase were injected using Fountain infusion systems (Merit Medical Systems, UT, USA) after intravenous injection of 5000 units of heparin. Another patient (Patient No. 4) had the arterial thromboses in his ulnar artery at the end of the session in his second treatment. Heparinized saline was injected from the catheter for 10 min. The treatment was successful in both patients. The skin ulcer in one patient (Patient No. 4) showed spontaneous healing on his visit to our outpatient clinic 20 weeks after the procedure.

The original signs and symptoms caused by AVM including bleeding, pain and swelling were improved. There were no major complications.

## Discussion

AVM is composed of feeding arteries, arteriovenous (AV) fistulae and draining veins. The target point of treatment is the fistula and its obliteration is essential. The AV fistula can be approached by three routes: transarterial, direct puncture and transvenous approaches [10]. Transarterial embolization has been performed with various materials, and sclerotherapy either percutaneously or transvenously for AVMs.

Transarterial embolization has been performed using diverse particulate agents including polyvinyl alcohol (PVA) [12, 13], trisacryl gelatin microspheres [14] and superabsorbent polymer (SAP) microspheres [15]. Despite the symptomatic relief provided by these particles, most lesions eventually recur because of the tendency of such particles to occlude proximal to the nidus of the AVM. This makes possible formation of a collateral blood supply to the nidus.

NBCA has been used for embolization of AVMs by selective catheterization or by direct puncture [16–18]. When NBCA is injected into the feeding artery of an AVM, it makes a cast filling the feeding artery and nidus. NBCA alone, however, is frequently insufficient to achieve complete occlusion of the AVM, especially when multiple fistulas are present. Because no convincing evidence has suggested permanent damage to the endothelium, NBCA is recommended for use in various aspects of the treatment of AVMs such as flow reduction or control of bleeding [19].

Absolute ethanol induces intimal denudation and rapid thrombosis. This may result in transmural vessel necrosis and diffusion into the surrounding tissues in some cases. Its effect on the vessel results in a permanent occlusion. However, ethanol injection is associated with pain, and serious complications including life-threatening cardiopulmonary collapse have been reported [20, 21].

Polidocanol works by damaging the endothelium lining inside of the blood vessels. This causes platelets and cellular debris to attach to the lining culminating in clotting of blood vessels. Over time, the clotted vein will be replaced with tissue [23]. Polidocanol is widely used for sclerotherapy of varicose veins in the lower extremities and esophageal varices. The sclerosing effect of polidocanol is more moderate than that of ethanol [24]. The maximum dose of polidocanol has been recommended as 2 mg/kg (0.067 mL/kg for 3% solution) because sinus arrest has been reported when a high dose is injected [23]. Polidocanol has been used for venous malformations [8, 9], with only a limited experience reported of its use for AVM. Ergun et al. [25] treated 10 AVMs with intraarterial polidocanol injection. Polidocanol was used alone or in combination with other agents, including NBCA, ethanol or coils. No major complications occurred and satisfactory results were assessed in all patients. No study has reported a larger number of cases using polidocanol.

We performed arterial embolization prior to sclerotherapy to promote stasis of polidocanol within the AVM. Performing sclerotherapy under sufficiently reduced blood flow will result in more prolonged stasis of the sclerosant enhancing the therapeutic effect and, at the same time, the possibility of reducing the volume of sclerosant needed. We used NBCA mostly to securely occlude the feeding arteries of AVM while avoiding the normal tissues.

The AVMs were classified according to the modified angiographic classification of Cho et al. [10] who found ethanol embolization to be most effective for type II (100%), and more effective for type IIIb (83%) than for type IIIa or mixed types (50%). In contrast, in our study of polidocanol sclerotherapy together with NBCA embolization, the efficacy did not show any significant difference according to the type of AVM. Our treatment method may thus be equally effective for all types of AVM, although additional larger studies will be necessary to confirm this.

When a draining vein was punctured in cases AV shunt was not dilated itself, we injected polidocanol under flow control to make the sclerosant retrograde to AV shunts.

Our result was comparable or better in comparison with the other therapeutic methods for AVM thus far reported in the literature. Only 8.3% of our patients experienced minor complications. This rate was lower compared with those in earlier investigations documenting the safety of polidocanol (Table 3). Polidocanol causes chemical damage to the vascular wall thereby inducing thrombosis, but unlike ethanol has little effect on the deep vascular layer and causes no penetrative damage [19]. Moreover, polidocanol sclerotherapy may be even safer than ethanol sclerotherapy with regard to nerve, skin and soft tissue damage.

**Table 3:** Summary of reports on treatment of AVMs

References	Method of treatment	No. of patients	Region of AVMs	Technical success (%)	Symptom improvement (%)	Complications (%)
Park [5]	Ethanol embolization	176	Extracranial	100	91	45
Osuga 2002 [15]	Microsphere embolization	23	Extracranial	100	91.3	0.4
Han [17]	NBCA embolization	14	Craniofacial	100	NA (resected)	0
Kaji [22]	NBCA, gelatin sponge embolization, ethanolamine oleate sclerotherapy	23	Extracranial	100	59.1	65.2
Kitagawa [6]	NBCA embolization, ethanolamine oleate sclerotherapy	24	Extracranial	100	83	16
Present study	NBCA embolization, polidocanol sclerotherapy	23	Extracranial	100	87	8.7

Several limitations can be noted in this study. First, it was conducted in a retrospective manner focusing on a relatively small number of cases. Second, the cases had involvement of diverse regions including head and neck, extremities and trunk precluding any precise comparative analysis with previous reports because of the different distribution of lesions studied in them. Third, not only the effectiveness but also the safety of polidocanol has yet to be sufficiently well documented in comparison with nonethanol sclerosing agents such as sodium tetradecyl sulfate (STS). Finally, further study will be needed to determine whether substantial reduction in the volume of polidocanol will be possible by taking advantage of the combination of sclerotherapy and transarterial embolization.

In conclusion, polidocanol sclerotherapy combined with transarterial embolization using NBCA is safe and effective for treating extracranial AVMs with an acceptable risk of minor complications.
